# Missed uterine rupture after vaginal delivery: a rare case of delayed diagnosis leading to sepsis and intra-abdominal abscess

**DOI:** 10.1093/jscr/rjaf291

**Published:** 2025-05-05

**Authors:** Mutasem Iqnaibi, Lana Sweity, Yomna Hroub, Bayan Saya’ra, Alaa R Al-Ihribat

**Affiliations:** College of Medicine and Health Sciences, Palestine Polytechnic University, Wadi Al-Hariyah Street, Hebron, 00970, State of Palestine; Princess Alia Governmental Hospital, Hebron, State of Palestine; College of Medicine and Health Sciences, Palestine Polytechnic University, Wadi Al-Hariyah Street, Hebron, 00970, State of Palestine; College of Medicine and Health Sciences, Palestine Polytechnic University, Wadi Al-Hariyah Street, Hebron, 00970, State of Palestine; College of Medicine and Health Sciences, Palestine Polytechnic University, Wadi Al-Hariyah Street, Hebron, 00970, State of Palestine; College of Medicine and Health Sciences, Palestine Polytechnic University, Wadi Al-Hariyah Street, Hebron, 00970, State of Palestine

**Keywords:** uterine rupture, silent rupture, cesarean scar, postpartum sepsis, vaginal delivery complication

## Abstract

Uterine rupture during pregnancy is a rare but life-threatening complication that poses severe risks to both mother and fetus. While it is typically associated with a history of uterine surgery, its silent presentation post-vaginal delivery remains exceptionally uncommon and diagnostically challenging. We present an extraordinary case of a 30-year-old multigravida at 33 + 2 weeks gestation, who developed a silent uterine rupture days after an uneventful vaginal delivery. The condition was masked by atypical symptoms, leading to delayed diagnosis, prolonged sepsis, and an intra-abdominal abscess. Despite the absence of classical warning signs, the rupture necessitated urgent surgical intervention, ultimately preserving maternal health and uterine integrity with loss of the fetus. This case underscores the critical need for heightened clinical suspicion and advanced imaging techniques in managing post-delivery complications, especially in patients with a scarred uterus.

## Introduction

Uterine rupture is a rare but devastating obstetric emergency that significantly contributes to maternal and neonatal morbidity [1]. While most cases occur during labor, rupture following an apparently uncomplicated vaginal delivery is an exceedingly rare phenomenon, often overlooked due to its subtle clinical presentation.

Population-based studies report an incidence of uterine rupture at 3.3 per 10 000 deliveries, with a strikingly higher rate of 22 per 10 000 among women with a prior cesarean section—highlighting uterine scarring as a predominant risk factor [2, 3]. However, postpartum silent rupture remains largely undocumented, making its recognition and timely intervention particularly challenging.

Management strategies vary from conservative monitoring to emergent laparotomy, depending on the severity of rupture and its complications. In cases of delayed diagnosis, secondary complications such as sepsis, abscess formation, and hemodynamic instability can necessitate urgent surgical repair or even hysterectomy, jeopardizing future fertility. Our case demonstrates how a silent rupture progressed insidiously, evading detection until advanced complications emerged.

## Case presentation

A 30-year-old multigravida (G6P5) at 33 + 2 weeks gestation presented with progressive, diffuse abdominal pain radiating to the back for 5 days. She had no chronic medical conditions but had a history of one prior cesarean section, with all subsequent deliveries being vaginal.

On arrival, she appeared pale, tachypneic, and clinically unwell. Abdominal examination revealed generalized tenderness without peritoneal signs or palpable contractions. She also reported burning micturition and dark-colored urine, raising concerns for a possible urinary tract infection or sepsis.

### Initial workup & misleading findings

Cardiotocography revealed a normoreactive fetal heart rate with mild, irregular uterine contractions. Laboratory investigations showed markedly elevated inflammatory markers [C-reactive protein (CRP), erythrocyte sedimentation rate (ESR)], prompting empiric antibiotic therapy and intravenous hydration. The elevated CRP and ESR were likely indicative of an ongoing inflammatory process, possibly early stage peritonitis or intra-abdominal infection, both of which became clinically evident in later stages.

Ultrasound imaging failed to detect any uterine rupture, placental abruption, or fetal compromise. The fetus appeared hemodynamically stable, with growth parameters consistent with gestational age:

- Biparietal diameter: 35 mm
- Head circumference: 32 + 1 mm
- Abdominal circumference: 33 + 6 mm
- Femur length: 33 + 2 mm
- Estimated fetal weight: 2300 g

### Rapid clinical deterioration

Three hours post-admission, the patient attempted to stand and suddenly experienced excruciating abdominal pain, followed by a sharp deceleration in fetal heart rate. Despite oxytocin augmentation, fetal demise occurred.

Over the next 10 h, she developed progressive pallor and hypotension, though remarkably, there were no signs of active bleeding—further masking the underlying uterine rupture. Conservative management continued with analgesics, likely further blunting classical symptoms and delaying diagnosis.

### Delayed recognition & surgical intervention

**Figure 1:**
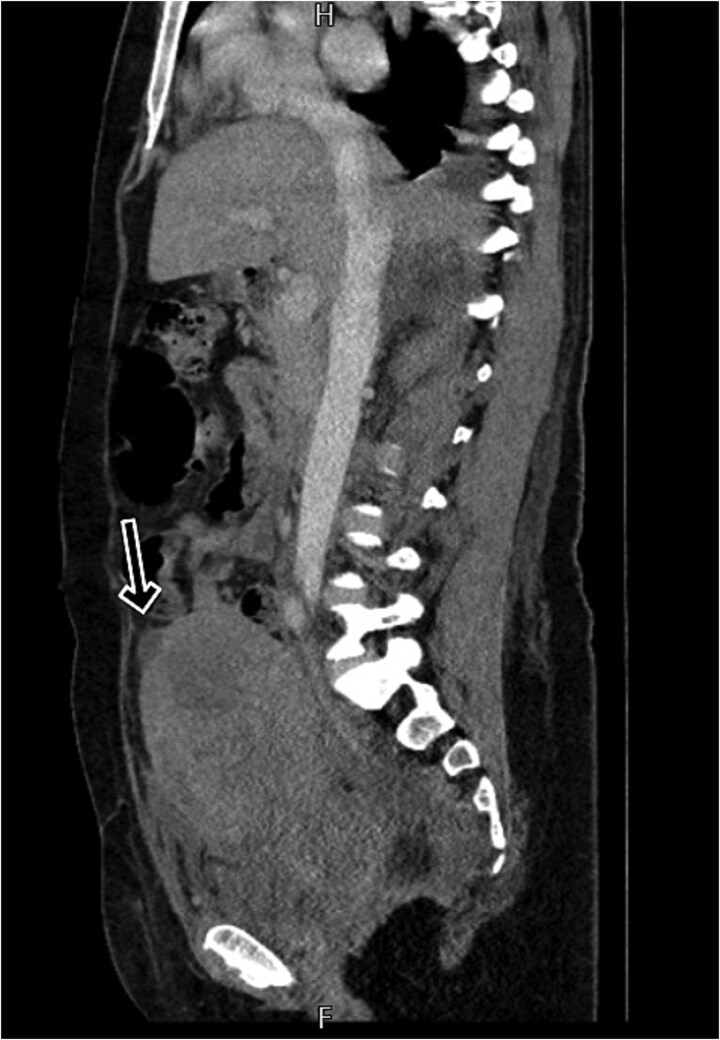
CT scan shows a linear small defect in the anterior wall of uterine fundus with small collection (2 × 1 cm).

**Figure 2:**
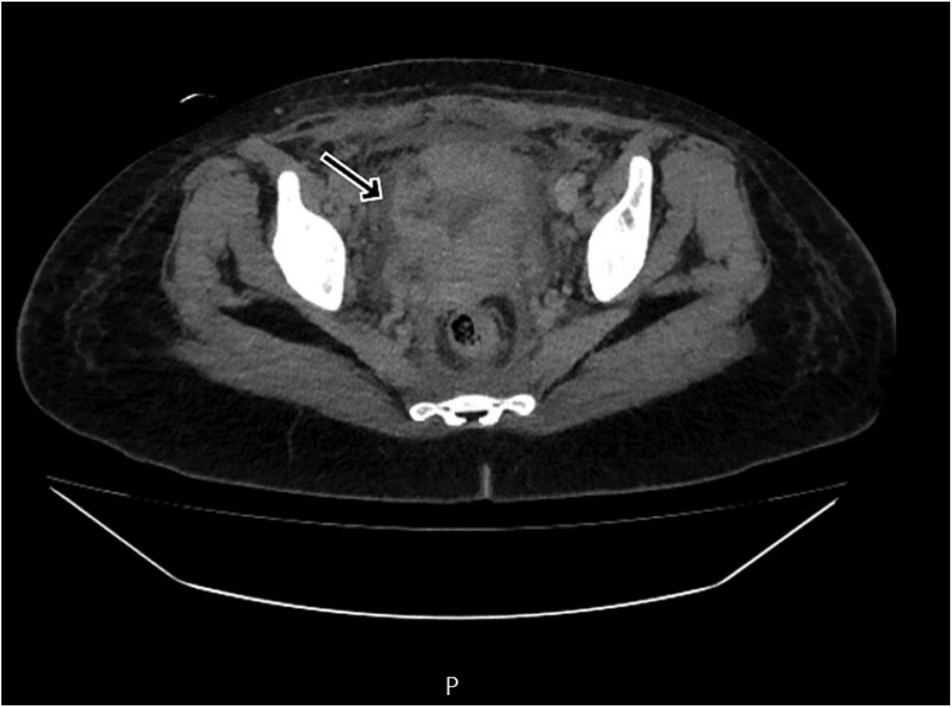
CT scan revealing a ruptured uterine scar with omental herniation.

By postpartum day five, the patient exhibited persistent fever and worsening clinical status, unresponsive to broad-spectrum antibiotics. A contrast-enhanced computed tomography (CT) scan was performed, revealing a ruptured uterine scar with omental herniation and a large intra-abdominal abscess (Figs 1 and 2).

An urgent exploratory laparotomy was performed, confirming a silent uterine rupture at the site of her previous cesarean scar. Although intraoperative images were not available, operative findings included omental herniation through the uterine defect and a localized intra-abdominal abscess. Surgical repair was successfully conducted under general anesthesia, with an estimated blood loss of 400 mL. The patient recovered uneventfully and was discharged on postpartum day 10 with preserved uterine function.

## Discussion

Uterine rupture is a catastrophic obstetric event characterized by a full-thickness tear in the uterine wall, leading to potential expulsion of the fetus into the peritoneal cavity. This condition poses significant risks, including severe maternal hemorrhage, fetal distress, and, in some cases, mortality. While uterine rupture predominantly occurs during labor [4], its manifestation in the postpartum period, especially following an unremarkable vaginal delivery, is exceedingly rare and often eludes prompt diagnosis.

The primary risk factor for uterine rupture is a scarred uterus, most commonly from a previous cesarean section [3]. Additional risk factors include high parity, uterine overdistension (as seen in multiple gestations or polyhydramnios), labor induction or augmentation with oxytocin, and a short interpregnancy interval [5]. Clinically, uterine rupture often presents with sudden, severe abdominal pain, vaginal bleeding, loss of fetal station, and signs of maternal shock [6, 7]. However, in rare instances, such as silent or subacute ruptures, these hallmark signs may be absent, leading to delayed recognition and management.

Timely diagnosis of uterine rupture necessitates a high index of suspicion, particularly in at-risk populations. Physical examination may reveal abdominal tenderness, uterine tenderness, or abnormal fetal heart patterns. Imaging modalities, including ultrasound and, in certain cases, CT [8], can aid in identifying uterine defects, hemoperitoneum, or fetal parts outside the uterine cavity. Nevertheless, imaging findings can sometimes be inconclusive, underscoring the importance of clinical vigilance.​

Our patient’s case is distinguished by the occurrence of a silent uterine rupture in the postpartum period, following an uneventful vaginal delivery at 33 + 2 weeks gestation. The absence of classic symptoms, coupled with nonspecific signs such as diffuse abdominal pain and elevated inflammatory markers, contributed to a delayed diagnosis. Notably, the rupture was identified only after the development of sepsis and an intra-abdominal abscess, five days postpartum, highlighting the atypical and insidious nature of this presentation.

Uterine rupture after one cesarean section followed by multiple vaginal deliveries is rare. Nam *et al.* [9] reported rupture during labor in a woman with two prior vaginal births after cesarean (VBAC), while Arusi *et al.* [10] linked prolonged labor and high fetal weight to increased risk. Our case differs in its silent, postpartum presentation without typical signs. Shah *et al.* [11] reported a rupture with abdominal pain and hemoperitoneum evident within hours of delivery, unlike our delayed presentation. Ahmed *et al.* [12] described uterine rupture presenting as paralytic ileus on day one postpartum, whereas our patient developed sepsis and abscess by day five. Elevated CRP and ESR in our patient likely reflected early inflammation or infection, highlighting their value as early markers in atypical postpartum cases [13].

## Conclusion

This case underscores the necessity for heightened clinical awareness regarding the potential for silent uterine rupture, even in patients with a history of successful VBACs. The atypical presentation and delayed diagnosis observed in our patient highlight the critical role of vigilant monitoring and consideration of uterine rupture in the differential diagnosis when encountering unexplained postpartum abdominal pain and sepsis.

## Data Availability

The data that support the findings of this study are available from corresponding author upon reasonable request.
